# Prognostic Value of New Sarcopenia Screening Tool in the Elderly—SARC-GLOBAL

**DOI:** 10.3390/nu16111717

**Published:** 2024-05-31

**Authors:** Ana Carolina Costa Vicedomini, Dan L. Waitzberg, Natalia Correia Lopes, Natalia Magalhães, Ana Paula A. Prudêncio, Wilson Jacob Filho, Alexandre Leopold Busse, Douglas Ferdinando, Tatiana Pereira Alves, Rosa Maria Rodrigues Pereira, Giliane Belarmino

**Affiliations:** 1 Department of Gastroenterology (LIM-35), School of Medicine, University of São Paulo, São Paulo 05508-220, Brazil; dan.waitzberg@gmail.com (D.L.W.); lopes.nataliac@gmail.com (N.C.L.); natalia.magalhaesnutri@hotmail.com (N.M.); apprudencio@gmail.com (A.P.A.P.); giliane85@hotmail.com (G.B.); 2 Medical Research Laboratory in Aging (LIM-66), School of Medicine, University of São Paulo, São Paulo 05508-220, Brazil; wiljac@usp.br (W.J.F.); a.busse@fm.usp.br (A.L.B.); ferdinando@usp.br (D.F.); tatiana.p.alves@gmail.com (T.P.A.); 3 Department of Research Laboratory in Rheumatology (LIM-17), School of Medicine, University of São Paulo, São Paulo 05508-220, Brazil; rosa.pereira@fm.usp.br

**Keywords:** sarcopenia, SARC-GLOBAL, SARC-F, SARC-CalF, EWGSOP2, clinical outcomes

## Abstract

Sarcopenia screening tools have a low capacity to predict adverse outcomes that are consequences of sarcopenia in the elderly population. This study aimed to evaluate the ability of a new sarcopenia screening tool SARC-GLOBAL to predict negative clinical outcomes in the elderly. A total of 395 individuals were evaluated in a 42-month period. The screening tools SARC-GLOBAL, SARC-F, and SARC-CalF and the diagnosis of sarcopenia according to European Working Group on Sarcopenia in Older Persons (EWGSOP2) were performed at the beginning of the study. Logistic and Poisson regression models were applied to assess the predictive value of the tools for the odds and risks of negative clinical outcomes, respectively. The most common negative clinical outcome in the followed population was falls (12.9%), followed by infections (12.4%), hospitalizations (11.8%), fractures (4.3%), and deaths (2.7%). Both SARC-GLOBAL and SARC-F were similar in predicting the odds of falls and hospitalizations during the follow up period, however SARC-CalF only predicted the odds of hospitalizations at 42 months.

## 1. Introduction

Aging is responsible for morphological, physiological and body composition changes. Among these alterations, we highlight the progressive reduction of muscle mass, reaching losses between 20 and 40% after the age of 70. These losses impact the reduction of strength and function, contributing to a functional decline with impact on the quality of life of these individuals [1,2,3,4].

This phenomenon is known as sarcopenia, it was first described by Rosenberg et al. in 1989, and is currently recognized as a muscular pathology by international disease code ICD-10-M62.5. Its prevalence can vary between 2% and 37% in the elderly and has potentially harmful consequences for individual and public health [5,6,7,8,9,10]. Negative outcomes have been reported as consequence of sarcopenia, such as changes in cognition, depression, loss of functional capacity, decreased quality of life, falls, hospitalizations, and mortality [11].

There is no universal consensus on the ideal evaluation methods and diagnostic criteria for sarcopenia in the elderly [12,13]. The model adopted and accepted in the literature is the European Consensus for the Definition and Diagnosis of Sarcopenia (European Working Group on Sarcopenia in Older People—EWGSOP2), which indicates that the diagnosis of sarcopenia should be based on sarcopenia screening, assessment of muscle strength, assessment of muscle mass, and the severity of sarcopenia through physical performance [13]. However, the proposed methods for sarcopenia diagnosis are onerous, which makes it unlikely to be reproduced in clinical practice and sarcopenia can be underdiagnosed, especially in places with low resources. Alternatives such as ultrasound have become scientific and can offer a viable diagnostic option due to their good association with other parameters related to sarcopenia, such as grip strength and skills [14]. This fact encourages the use of screening tools, such as the sarcopenia screening tool SARC-F and SARC-CalF questionnaires, however both tools have a low capacity to predict adverse outcomes that are consequences of sarcopenia in the elderly population [15,16].

A good sarcopenia screening tool is therefore considered valuable for predicting these clinical outcomes and leading to early interventions. Therefore, our objective was to evaluate the ability of a new sarcopenia screening tool SARC-GLOBAL to predict negative clinical outcomes (falls, fractures, infections, hospitalizations, and deaths) in the elderly in the 42-month period after its application.

## 2. Materials and Methods

### 2.1. Study Design and Participants

The study was a prospective longitudinal analysis of a large study designed to validate a new sarcopenia screening tool in the elderly. The volunteers were selected from the community and from the geriatric outpatient clinic of the Hospital das Clínicas of the Federal University of São Paulo. Between February 2016 and December 2019, 405 volunteers were recruited and 395 included. Inclusion criteria were age ≥ 60 years, non-institutionalized, and able to consciously answer questions asked in the anamnesis, sarcopenia screening questionnaires, and cognitive questionnaire. We excluded individuals under 60 years old, and those with physical disability and dementia. A single trained technician performed all the study assessments, which were done respecting the ethical standards of the Declaration of Helsinki of the World Medical Association and after participant’s signature of an informed consent. The study protocol was approved by the Institutional Ethics Review Board (1.905.072) and registered at www.clinicalTrials.gov https://clinicaltrials.gov/study/NCT04451005?cond=(NCT04451005)&rank=1 (accessed on 22 May 2024, NCT04451005).

### 2.2. Participant Information

Participants’ demographic information, lifestyle variables, and personal disease history were collected. The variables included age, sex, history of previous illnesses, and regular physical activity. To assess physical activity, the International Physical Activity Questionnaire (IPAQ) was performed in the form of an interview [17].

Self-perception on the general health status was assessed through the question: “How do you rate your health, excellent, good, regular, bad or terrible”? [8].

### 2.3. Anthropometric Measurements

Anthropometric parameters included height, weight, and calf circumference (CC). Height and weight were measured while the participants were barefoot and in light clothing using the height and weight scale to the nearest 0.1 cm and 0.1 kg, respectively. Body mass index (BMI) was calculated as weight in kilograms divided by height in meters squared. CC was measured with millimeter non-elastic tape, with the with the elderly individual in standing position, at the greatest circumference of the lower right leg, and recorded in centimeters (cm), accurate to one decimal place. All measurements were performed in duplicate, and the means were calculated for analysis.

### 2.4. Sarcopenia Screening

Sarcopenia screening was performed using the SARC-F, SARC-CalF, and SARC-GLOBAL tools [15,16]. The three questionnaires were applied by a single trained evaluator through face-to-face interviews.

The SARC-F includes 5 items, namely strength, assistance in walking, getting up from a chair, climbing stairs, and falls, while the SARC-CalF has the additional calf circumference (CC). Total SARC-F score ≥ 4 and total SARC-CafF score ≥ 11 indicate possible sarcopenia [15,16].

The SARC-GLOBAL was applied in two stages. The first stage includes 5 items, namely strength, assistance for walking, getting up from a chair, climbing stairs, and falls. The second stage was collected global data of the elderly as follows: sex, age, medication in use, body mass index (BMI), hand grip strength (HGS), arm circumference (AC), and calf circumference (CC). The score raises from 0 to 26 points, with two possible classifications, from 0–10: Healthy patient, with no indication of sarcopenia and from 11–26: a probable diagnosis of sarcopenia 18.

### 2.5. Diagnosis of Sarcopenia

The diagnosis of sarcopenia was performed considering the method proposed by the criteria established by the European Working Group on Sarcopenia in Older Persons (EWGSOP2), which considers sarcopenia as the loss of lean mass, accompanied by loss of strength and function [13].

#### Muscle Mass, Strength, and Performance

At T0, we calculated the appendicular skeletal mass index (ASMI) as a marker of skeletal muscle mass (SMM), as described elsewhere 9. Briefly, this was done by summing the lean mass of the four limbs, obtained by dual-energy x-ray absorptiometry (DXA), and applying the following equation: ASMI = appendicular skeletal mass/height^2^ (kg/m^2^) [10]. The hand grip strength (HGS) was assessed as a marker of SMM strength by using an analog dynamometer (JAMAR^®^ Sammons Preston, Inc. (Warrenville, IL, USA^)^). During the analysis, the subjects remained seated in a height-adjustable chair, with their legs upright and their feet flat on the floor, to obtain a right angle in the hip, knee, and ankle joints. The test arm remained close to the body, with the elbow flexed in a 90° position, with the palm facing towards the body, and the thumb pointing upwards. The arm that was not being evaluated remained supported and relaxed on the thigh [10,11]. The walking speed was assessed as a marker of SMM performance by measuring the time expended to cover 4 or 6 m [10,11]. Participants were advised to be fasting for 4 h and to refrain from physical activity (except light walking), diuretic use, and alcohol consumption for 24 h before these assessments.

### 2.6. Follow-Up for Adverse Outcomes

**Figure 1:**
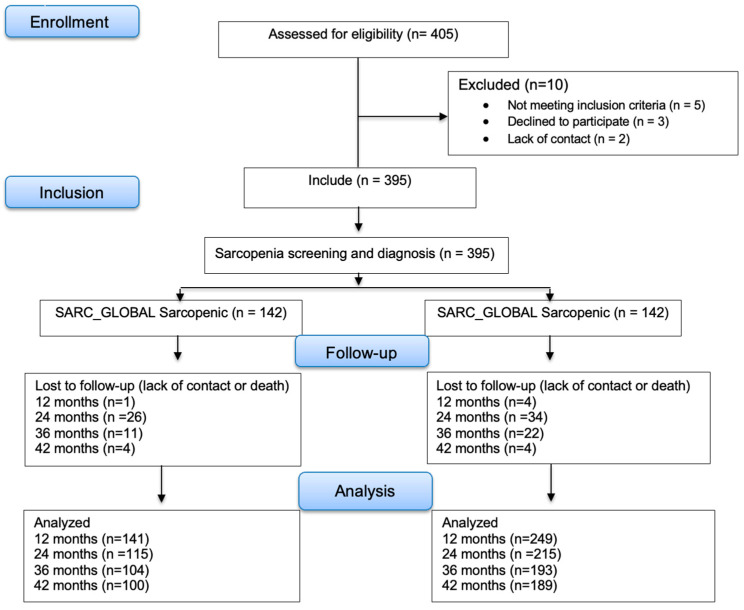
CONSORT flow diagram of study population and analysis.

All the participants were followed by telephone contact carried out by a single survey annually during the period of 42 months. This follow-up assessed the occurrence of negative clinical outcomes including falls, fractures, infections, hospitalizations, and mortality, as well as the practice of physical activity and use of oral supplements. All deaths occurring in the study period were included. During the follow-up period we excluded patients due to the lack of telephone contact (Figure 1).

### 2.7. Statistical Analysis

Continuous variables were presented as mean ± standard deviation and the differences between sarcopenic and non-sarcopenic participants were evaluated with the Mann–Whitney U test or Brunner Munzel test. Categorical variables were presented as absolute and relative frequencies, and sarcopenic and non-sarcopenic participants comparisons were analyzed using the Fisher’s Exact Test.

The predictive performance of sarcopenia screening tools for clinical outcomes was assessed by regression models. Logistic regression models were used for categorical/binary outcome variables. The zero-inflated Poisson model was used for continuous variables of clinical outcomes, due to the large number of zeros (for example, no outcome registered). Odds Ratio (OR) and Relative Risk (RR) at 95% confidence intervals (95% CI) from logistic regression and Poisson models, respectively, were used to estimate the association of sarcopenia with clinical outcomes. 

The data were analyzed using R software (version 4.2). The significance level adopted in the tests was 95% (*p* < 0.05) and two-tailed hypotheses were considered. 

## 3. Results

### 3.1. Study Population Characteristics

The sample characteristics are presented in Table 1. A total of 395 individuals met the eligibility criteria and completed the follow-up period of the study. The average age was 70.7 ± 7.5 years, including 19% men and 81% women. 

The most common negative clinical outcome In the followed population was falls (12.9%), followed by infections (12.4%), hospitalizations (11.8%), fractures (4.3%), and deaths (2.7%). As shown in Table 2, negative clinical outcomes were evaluated according to SARC-GLOBAL sarcopenia screening tool. The findings for the other tools, EWGSOP2, SARC-F, and SARC-CalF, are presented in Supplementary Table S1.

### 3.2. Predictive Performance of the of SARC-GLOBAL, SARC-F, and SARC-CalF Sarcopenia Screening Tools for Clinical Outcomes According to Logistic Regression

The predictive performance of the of SARC-GLOBAL, SARC-F, and SARC-CalF Sarcopenia screening tools for clinical outcomes according to logistic regression are presented in Table 3. We performed logistic regression models to investigate the chances (odds) of the sarcopenia screening tools to predict binary outcomes (yes or no). 

During all periods of the follow-up time, SARC-GLOBAL tool showed predictive value for chance of falls (*p* < 0.05). Also, it was able to predict the odds of hospitalizations at 24 months [OR 1.81 (CI 1.06–3.10); *p* = 0.031)], 36 months [OR 1.82 (CI 1.07–3.10)]; *p* = 0.027), and 42 months [OR 1.86 (CI 1.14–3.04); *p* = 0.013)]. 

Similar to the SARC-GLOBAL, the SARC-F tool was also able to predict the chance of falls, except at 24 months, and hospitalizations during all the follow-up time (Table 3). On the other hand, the SARC-CalF tool did not predict the odds of falls, and only predicted the odds of hospitalizations at 42 months [OR 1.94 (1.03–3.66); *p* = 0.041)].

In contrast, sarcopenia evaluated by the EWGSOP2 did not predict the odds for any of the clinical outcomes evaluated (*p* > 0.05). The odds of fracture and infection outcomes were not predicted by all the screening tools (*p* > 0.05). 

Due to the small number of deaths in the follow up period it was not possible to establish regression models for the mortality outcome. 

### 3.3. Predictive Performance of the of SARC-GLOBAL, SARC-F, and SARC-CalF Sarcopenia Screening Tools for Clinical Outcomes according to Poisson Regression

To assess the predictive value of sarcopenia screening tools for estimation of risk for clinical outcomes we performed Poisson regression models, as described in Table 4. We performed Poisson regression models to investigate the risk of the sarcopenia screening tools to predict the incidence of the outcomes that occurred during the follow-up period. 

Sarcopenia screening using the SARC-GLOBAL tool was able to predict the risk of falls and hospitalization at 12, 24, 36, and 42 months of follow-up (*p* < 0.007). Also, SARC-GLOBAL was the only screening tool able to predict the risk of infections at 24 [RR 1.43 (CI 1.31–2.76); *p* < 0.001)], 36 [RR 1.95 (CI 1.34–2.85); *p* < 0.001)], and 42 months [RR 1.70 (CI 1.25–2.31); *p* < 0.001)] of follow-up. 

Similar to the SARC-GLOBAL, the SARC-F tool was also able to predict the risk of falls and hospitalizations during the follow-up time (Table 3), however it could not predict the risk of infections (*p* > 0.05). On the other hand, the SARC-CalF tool did not predict the risk of falls, although it did predict the risk of infections at 24 [RR 1.60 (CI 1.00–2.55); *p* = 0.049)] and 36 months [RR 1.70 (CI 1.07–2.70); *p* = 0.024)] and the risk of hospitalizations during all the follow-up periods (*p* < 0.03).

In contrast, the only outcome predicted by the EWGSOP2 during all the follow up was the risk of infections (*p* < 0.04). The risk of fracture was not predicted by any of the screening tools (*p* > 0.05).

## 4. Discussion

The findings of our study reveal that, according to the SARC-GLOBAL tool, 35.9% of the participants had a probable diagnosis of sarcopenia, with 21.5% confirmed by the EWGSOP2 criteria. The negative clinical outcomes observed during the follow-up period were falls, infections, hospitalizations, fractures, and deaths. Among the elderly with a probable diagnosis of sarcopenia as per SARC-GLOBAL, there was a higher incidence of falls, hospitalizations, and deaths compared to the non-sarcopenic group It is well established that sarcopenia is associated with negative health-related outcomes [11,18,19,20,21]. The pathophysiological bases of sarcopenia, including neuromuscular degeneration, alterations in muscle protein turnover, changes in hormone levels and insulin sensitivity, behavioral/lifestyle factors, and the presence of chronic inflammation, increases the individual’s susceptibility to negative outcomes [19,20,21,22,23]. 

In addition, our study demonstrated that sarcopenic screening tools can predict negative clinical outcomes in the elderly population. The predictive performance varied between the SARC-GLOBAL, SARC-F, and SARC-CalF tools as per logistic regression. Both SARC-GLOBAL and SARC-F were similarly effective in predicting the odds of falls and hospitalizations during the follow up period. However, the SARC-CalF tool only predicted the odds of hospitalizations at 42 months. We found both SARC-GLOBAL and SARC-F to have a strong predictive value for the occurrence of falls and hospitalization, given that both questionnaires contain items related to falls and mobility. Thus, the chances of hospitalization have been linked with immobilization and inactivity [24]. Despite SARC-CalF also incorporating questions about falls and mobility, the calf circumference carries a higher score value among the other items, and the elderly participants evaluated were mainly overweight, which could overestimate calf circumference. Hence, this could have constrained SARC-CalF’s ability to predict the odds of falls and hospitalization occurrence. 

On the other hand, to evaluate the sarcopenia screening tool’s estimation of the risks number of each negative outcome, we utilized Poisson regression models. These models were better suited to our data as we evaluated the number of the incidence of the outcomes in months of follow up. Consequently, all three tools proved to be robust instruments for predictions on the risk of the number of hospitalizations that occurred during all the follow-up periods. Moreover, SARC-GLOBAL and SARC-F were similar in predicting the risk of incidence of falls during the follow up period, just as SARC-GLOBAL and SARC-CalF were for predicting the risk of infection incidences. However, it is noteworthy that SARC-GLOBAL was the only screening tool capable of predicting the risk of the number of infections at three of the four time periods of the follow-up. Also, different from SARC-GLOBAL, SARC-CalF did not predict the risk of the incidence of falls [25].

The robust predictive value of SARC-GLOBAL and SARC-F for the risk of the incidence of falls can be explained by the items related to falls and mobility in both questionnaires, as previously discussed. Also, the absence of a predictive value of SARC-CalF for this outcome could be linked to the high score for calf circumference attributed to overweight participants. The risk for the incidence of infection was predicted by both SARC-GLOBAL and SARC-CalF, although SARC-GLOBAL projected this over a longer term (42 months). The susceptibility for infections among the elderly is associate with the presence of sarcopenia, comorbidities, and polypharmacy [26]. Thus, anthropometric measures related to muscle mass in both tools, and the variables of global health of the elderly in SARC-GLOBAL, could explain our findings. 

Our results also indicate that sarcopenia evaluated by the EWGSOP2 criteria did not predict the odds for any of the clinical outcomes assessed and only predicted the risk of the number of infections during all follow-up periods. This is a notable finding considering numerous studies have associated sarcopenia diagnosis with negative outcomes. For instance, Yeung et al., 2019 performed a meta-analysis with prospective studies and showed that sarcopenia was associated with higher odds of falls (OR 1.89, 95% CI 1.33–2.68, *p* < 0.001) and fractures (OR 1.71, 95% CI 1.44–2.03, *p* = 0.011) [10]. Also, a prospective study evaluated 384 elderly and found that EWGSOP2 criteria were able to predict the incidence of falls (HR 1.86, 95% CI 1.22–1.84) in twelve months of follow up [27].

Based on our results we hypothesize that the odds (OR) and risks (RR) for hospitalizations and falls are better predicted from the SARC-GLOBAL and SARC-F tools than by the actual diagnosis of sarcopenia (EWGSOP2) given that these tools consider variables directly related to mobility. As demonstrated in the study by Schene et al. (2024) [28], who not only investigated sarcopenia, but also evaluated the association of physical performance in 1789 patients in a fracture center using several tools, and despite the low prevalence of sarcopenia (2.8%), revealed a low physical performance, mainly in patients with hip fractures, both men and women. These findings underscore the importance of considering physical performance assessment alongside muscle mass evaluation, highlighting the comprehensive nature of sarcopenia assessment in clinical practice [28]. 

In addition, SARC-GLOBAL includes important variables related to these outcomes such as age [29], medication in use [30], and BMI [31] which could provide a more holistic approach to predicting these outcomes.

According to the study conducted by Martone et al. [32] the incidence of sarcopenia during hospitalization was significantly associated with the number of days spent in bed but was not correlated with the total length of hospital stay. Specifically, patients who developed sarcopenia spent an average of 5.1 days bedridden, compared to 3.2 days for those without sarcopenia at discharge (*p* = 0.02). Patients with sarcopenia exhibited a significantly lower body mass index compared to non-sarcopenic counterparts (25.0 ± 3.8 kg/m² vs. 27.6 ± 4.9 kg/m², respectively; *p* < 0.001). These findings suggest that sarcopenia may play a significant role in the duration of bed rest during hospitalization, highlighting its clinical relevance as a risk factor for adverse outcomes in hospitalized patients [32].

Other research has evaluated the predictive value of SARC-F and SARC-CalF in various elderly population. For instance, a SARC-F score of 2 or higher significantly increased the hazard ratio for falls [HR 2.11 (CI 1.37–3.26), *p* < 0.001)] among hospitalized elderly individuals 33. SARC-F was also able to predict clinical outcomes for stroke patients [33]. The risk of sarcopenia as measured by SARC-F (aHR = 2.51; 95%CI: 1.40–2.77) and SARC-CalF (aHR = 2.04; 95%CI: 1.55–4.02) was associated with a higher risk of death in older men with cancer [34]. 

However, SARC-F was a better predictor of 1-year mortality (adjusted HR: 2.08; 95% CI: 1.27–3.42) in Chinese nursing home residents than SARC-CalF (adjusted HR: 1.54; 95% CI: 0.95–2.47) [35]. In addition, for hospitalized elderly, SARC-CalF was not found to be a predictor of clinical outcomes in the 6 months following discharge [36]. 

This study also highlighted that all three sarcopenia screening tools SARC-F, SARC-Calf, and SARC-GLOBAL, could not reliably predict the odds of fracture, mortality, and infections, or the risk of fracture and mortality in a 42-month period. These findings suggest that other factors beyond sarcopenia may be more influential in predicting these specific outcomes.

However, this study represents an important contribution to the field. To the best of our knowledge, this is the first study to investigate the ability of the novel SARC-GLOBAL sarcopenia screening tool to predict negative clinical outcomes in the elderly over a 42-month period. Our results demonstrate that, despite their shared features, each screening tool offers a unique predictive capacity.

Notably, the SARC-GLOBAL was the only one that simultaneously predicted the risk of falls, hospitalization, and infections, demonstrating its unique utility. Future research could further explore the mechanisms behind these predictions, as well as investigate ways to further refine and improve the accuracy of these sarcopenia screening tools. 

This study has some limitations: (1) The 4-year follow-up period may have limited the recording of a greater number of events; (2) A majority of participants included females, which means that the association in males may vary; (3) The study population consisted of patients from the outpatient clinic and from a center for the elderly, and the results of the study cannot be extrapolated to the general population; (4) We did not obtain records of laboratory tests, lifestyle, and nutritional status during the follow-up period; (5) Follow-up through telephone contact and the time period established between calls can be confusing factors for the studied population. 

Despite limitations, this study evaluated the ability of a new sarcopenia screening tool in the elderly, the SARC-GLOBAL, to predict negative clinical outcomes in the elderly. The reference method for diagnosing sarcopenia in clinical practice can be quite costly and difficult for professionals to access. Sarcopenia screening, identified by the SARC-GLOBAL tool, seems to be a good instrument with good screening capacity and seems to be better for predicting outcomes such as falls, hospitalizations, and infections in the elderly, compared to currently existing screening tools such as SARC-F and SARC-Calf. 

We acknowledge the limitations inherent in our study. The four-year follow-up period may have limited the recording of a greater number of events, and the predominance of female participants raises questions about the generalizability of our findings to males. Furthermore, the composition of our study population from outpatient clinics and centers for the elderly may restrict the extrapolation of our results to the broader general population. We also regret the absence of records regarding laboratory tests, lifestyle, and nutritional status during the follow-up period, which could have enriched our analysis. Despite these limitations, we remain committed to advancing scientific understanding in this area and will endeavor to address these challenges in future research efforts. By extending the duration of follow-up, diversifying study populations, and enhancing data collection methods, we aim to contribute to a more comprehensive understanding of sarcopenia and its implications for diverse populations.

## 5. Conclusions

The new sarcopenia screening tool SARC-GLOBAL was able to predict the chances of falls and hospitalizations and the risk of incidence of falls, hospitalizations, and infections in the elderly in the 42-month period after its application. The SARC-GLOBAL showed to be better to concomitantly predict the risk of falls, hospitalization, and infections when compared to other sarcopenia screening tools.

## Figures and Tables

**Table 1:** Demographic characteristics and clinical data of all the participants according to sarcopenia screening.

Variables	Total (*n* = 395)	SARC-GLOBAL Sarcopenic	SARC-GLOBAL Non-Sarcopenic	*p*-Value ^1^
Sex				
Female, n (%)	320 (81)	107 (75.3)	214 (84.6)	
Male, n (%)	75 (19)	35 (24.7)	39 (15.4)	0.031 ^2^
Age, years	70.7 ± 7.5	74.6 ± 8.2	68.5 ± 6.1	<0.001 ^3^
Comorbidities ^4^, n (%)	57 (14.4)	35 (14.7)	34 (24.6)	<0.001 ^5^
Medications ^6^, n (%)	199 (50.4)	102 (40.3)	97 (68.3)	<0.001 ^5^
Anthropometric data				
Weight, kg	67.8 ± 14.2	65.4 ± 15.3	69.2 ± 13.5	0.013 ^7^
BMI ^8^, kg/m^2^	27.6 ± 5.3	26.9 ± 5.9	28.0 ± 4.9	0.070 ^3^
Arm circumference, cm	32.0 ± 4.5	30.8 ± 4.9	32.9 ± 4.1	<0.001 ^3^
Calf circumference, cm	35.9 ± 3.6	34.9 ± 4.0	36.5 ± 3.1	<0.001 ^3^
ASMI ^9^, kg/m^2^	6.6 ± 0.2	6.4 ± 1.7	6.7 ± 1.6	0.003 ^7^
HGS ^10^	17.1 ± 7.2	12.9 ± 5.0	19.5 ± 7.1	<0.001 ^3^
EWGSOP2 ^11^				
Sarcopenic, n (%)	85 (21.5)	22 (8.7)	63 (44.4)	0.736 ^2^
Non-sarcopenic, n (%)	310 (78.5)	231 (91.3)	79 (55.6)	
SARC-GLOBAL, n (%)	NA ^12^	142 (35.9)	253 (64.1)	<0.001 ^5^ 0.001 ^2^
Physical practice, IPAC ^13^				
Sedentary, n (%)	53 (13.7)	24 (9.6)	29 (20.9)	
Irregularly active a, n (%)	121 (3.2)	73 (29.3)	48 (34.5)	
Irregularly active b, n (%)	146 (37.6)	98 (39.4)	48 (34.5)	
Active, n (%)	58 (15.0)	44 (17.7)	14 (10.1)	
Very active, n (%)	10 (2.6)	10 (4.0)	0 (0.0)	

^1^ *p*-value for comparison between sarcopenic and non-sarcopenic participants. ^2^ Fisher’s Exact Test. ^3^ Brunner Munzel test. ^4^, ≥five diseases. ^5^ Chi-Squared. ^6^ ≥four medications. ^7^ Mann–Whitney test. ^8^ BMI = Body Massa Index. ^9^ ASMI = appendicular skeletal mass index. ^10^ HGS = hand grip strength. ^11^ EWGSOP2 = European Working Group on Sarcopenia in Older Persons. ^12^ NA = Not applicable. ^13^ IPAC = International Physical Activity Questionnaire. Data are shown as the mean ± standard deviation or absolute (relative) frequencies.

**Table 2:** Outcomes observed at follow-up time according to sarcopenia screening.

Follow-Up Time (Months)	SARC-GLOBAL Sarcopenic				SARC-GLOBAL Non-Sarcopenic			
	12	24	36	42	12	24	36	42
Clinical Outcomes								
Falls	34 (24.1)	47 (40.9)	47 (45.2)	49 (49.0)	33 (12.3)	46 (21.4)	49 (25.4)	53 (28.0)
Factures	3 (2.1)	4 (3.6)	5 (5.2)	7 (7.9)	0 (0.0)	8 (3.8)	10 (5.4)	13 (7.4)
Infections	25 (17.7)	35 (30.4)	38 (35.9)	42 (42.0)	44 (17.7)	55 (25.0)	60 (30.2)	65 (33.9)
Hospitalizations	22 (15.6)	32 (28.1)	37 (35.6)	40 (40.4)	24 (9.6)	38 (17.8)	42 (21.9)	44 (23.8)
Death	3 (2.1)	6 (5.1)	8 (7.8)	9 (9.5)	4 (1.6)	1 (0.5)	2 (1.1)	2 (1.2)
Total	141	115	104	100	249	215	193	189

Data are shown as absolute (relative) frequencies.

**Table 3:** Logistic regression model for estimation of odds of falls, fractures, infections, and hospitalizations at 12, 24, 36, and 42 months in sarcopenic participants.

Follow-Up (Months)	SARC-GLOBAL			EWGSOP2 ^1^			SARC-F			SARC-CalF		
	β	OR (CI 95%)	*p*-Value ^2^	β	OR (CI 95%)	*p*-Value ^2^	β	OR (CI 95%)	*p*-Value ^2^	β	OR (CI 95%)	*p*-Value ^2^
Falls												
12	0.73	2.07 (1.22–3.54)	0.007	−0.06	0.94 (0.49–1.79)	0.845	1.48	4.40 (2.45–7.88)	<0.001	−0.02	0.98 (0.46–2.13)	0.967
24	0.93	2.54 (1.55–4.16)	<0.001	0.02	1.02 (0.57–1.84)	0.935	1.16	0.98 (0.48–2.01)	0.966	−0.02	0.98 (0.48–2.01)	0.966
36	0.97	2.64 (1.58–4.38)	<0.001	0.00	1.00 (0.55–1.81)	0.990	1.10	3.02 (1.68–5.41)	<0.001	−0.03	0.97 (0.47–2.01)	0.937
42	0.69	1.99 (1.26–3.15)	0.003	−0.08	0.93 (0.53–1.61)	0.791	1.09	2.98 (1.74–5.09)	<0.001	−0.20	0.82 (0.41–1.63)	0.570
Fractures												
12	18.73	>1000 (0-inf)	0.995	0.59	1.80 (0.16–20.13)	0.632	0.83	2.30 (0.21–25.77)	0.498	2.58	13.18 (1.17–147.9)	0.037
24	−0.07	0.93 (0.27–3.16)	0.908	−1.13	0.32 (0.04–2.54)	0.282	−0.95	0.39 (0.05–3.04)	0.366	0.27	1.31 (0.28–6.18)	0.735
36	0.06	1.06 (0.34–3.25)	0.923	−0.55	0.58 (0.13–2.64)	0.477	−1.19	0.30 (0.04–2.37)	0.255	0.56	1.75 (0.47–6.58)	0.407
42	−0.04	0.96 (0.37–2.46)	0.928	−0.94	0.39 (0.09–1.72)	0.214	−0.71	0.49 (0.11–2.17)	0.350	0.51	1.66 (0.53–5.18)	0.380
Infections												
12	0.00	1.00 (0.58–1.73)	0.988	0.10	1.10 (0.59–2.05)	0.757	−0.05	0.95 (0.48–1.89)	0.894	0.09	1.10 (0.52–2.30)	0.809
24	0.27	1.31 (0.80–2.17)	0.287	−1.13	0.32 (0.04–2.54)	0.282	0.13	1.14 (0.62–2.10)	0.670	0.40	1.49 (0.76–2.93)	0.248
36	0.28	1.33 (0.80–2.19)	0.269	−0.55	0.58 (0.13–2.64)	0.477	0.05	1.06 (0.57–1.94)	0.862	0.25	1.28 (0.64–2.54)	0.481
42	0.19	1.21 (0.77–1.92)	0.405	0.00	1.00 (0.58–1.71)	0.994	0.07	1.07 (0.60–1.89)	0.821	0.07	1.07 (0.56–2.04)	0.831
Hospitalizations												
12	0.55	1.73 (0.93–3.22)	0.082	0.27	0.13 (0.09–0.18)	0.454	0.81	2.25 (1.13–4.49)	0.021	0.51	1.66 (0.75–3.67)	0.212
24	0.59	1.81 (1.06–3.10)	0.031	0.29	1.34 (0.72–2.48)	0.353	0.98	2.65 (1.44–4.89)	0.002	0.51	1.67 (0.82–3.40)	0.157
36	0.60	1.82 (1.07–3.10)	0.027	0.13	1.14 (0.62–2.10)	0.666	0.81	2.25 (1.23–4.13)	0.009	0.55	1.73 (0.86–3.46)	0.121
42	0.62	1.86 (1.14–3.04)	0.013	0.25	1.29 (0.73–2.26)	0.382	0.97	2.65 (1.51- 4.64)	0.001	0.66	1.94 (1.03–3.66)	0.041

^1^ EWGSOP2 = European Working Group on Sarcopenia in Older Persons. ^2^ Logistic Regression, statistical significance *p* < 0.05. OR = Odds Ratio. CI = Confidence Interval.

**Table 4:** Poisson regression model for estimation of risk of falls, hospitalizations, fractures, infections at 12, 24, 36, and 42 months in sarcopenic participants.

Follow-Up (Months)	SARC-GLOBAL			EWGSOP2 ^1^			SARC-F			SARC-CalF		
	β	RR (CI 95%)	*p*-Value ^2^	β	RR (CI 95%)	*p*-Value ^2^	β	RR (CI 95%)	*p*-Value ^2^	β	RR (CI 95%)	*p*-Value ^2^
Fall												
12	0.61	1.84 (1.23–2.76)	0.003	−0.38	0.68 (0.39–1.18)	0.173	1.39	4.02 (2.68–6.03)	<0.001	−0.40	0.67 (0.34–1.34)	0.259
24	0.70	2.01 (1.44–2.81)	<0.001	−0.17	0.84 (0.55–1.29)	0.434	0.87	2.38 (1.68–3.37)	<0.001	−0.24	0.78 (0.46–1.34)	0.376
36	0.64	1.90 (1.35–2.66)	<0.001	−0.06	0.94 (0.62–1.42)	0.767	0.81	2.26 (1.59–3.21)	<0.001	−0.25	0.78 (0.46–1.33)	0.363
42	0.57	1.76 (1.32–2.36)	<0.001	0.18	1.19 (0.80–1.78)	0.386	0.97	2.65 (1.86–3.78)	<0.001	−0.01	0.99 (0.59–1.65)	0.966
Fractures												
12	18.74	>1000 (0-inf)	0.994	0.18	1.20 (0.12–11.50)	0.877	1.52	4.57 (0.64–32.45)	0.129	1.85	6.36 (0.90–45.14)	0.064
24	0.34	1.40 (0.49–4.03)	0.534	−1.27	0.28 (0.04–2.15)	0.221	−0.32	0.73 (0.16–3.24)	0.674	0.07	1.08 (0.24–4.81)	0.922
36	0.37	1.44 (0.50–4.16)	0.496	−0.56	0.57 (0.13–2.56)	0.465	−16.54	0.00 (0.00–inf)	0.990	0.04	1.04 (0.23–4.64)	0.960
42	0.34	1.40 (0.64–3.08)	0.404	−0.82	0.44 (0.10–1.92)	0.275	−1.41	0.24 (0.03–1.84)	0.171	0.24	1.27 (0.37–4.40)	0.702
Infections												
12	0.36	1.43 (0.95–2.14)	0.087	0.52	1.68 (1.09–2.60)	0.019	−0.06	0.94 (0.55–1.61)	0.815	0.34	1.40 (0.83–2.37)	0.206
24	0.64	1.90 (1.31–2.76)	<0.001	0.44	1.55 (1.03–2.32)	0.036	0.01	1.01 (0.63–1.63)	0.951	0.47	1.60 (1.00–2.55)	0.049
36	0.67	1.95 (1.34–2.85)	<0.001	0,59	1.81 (1.21–2.69)	0.004	0.16	1.18 (0.75–1.86)	0.480	0.53	1.70 (1.07–2.70)	0.024
42	0.53	1.70 (1.25–2.31)	<0.001	0.53	1.71 (1.16–2.50)	0.006	0.24	1.27 (0.83–1.94)	0.265	0.42	1.52 (0.96–2.39)	0.072
Hospitalizations												
12	0.77	2.15 (1.35–3.43)	0.001	0.48	1.61 (0.97–2.66)	0.063	0.65	1.92 (1.15–3.20)	0.012	0.84	2.32 (1.37–3.93)	0.002
24	0.73	2.08 (1.39–3.12)	<0.001	0.32	1.38 (0.88–2.17)	0.160	0.84	2.32 (1.52–3.55)	<0.001	0.79	2.19 (1.38–3.48)	<0.001
36	0.58	1.79 (1.17–2.72)	0.007	0.02	1.02 (0.62–1.68)	0.936	0.91	2.49 (1.61–3.84)	<0.001	0.55	1.74 (1.04–2.89)	0.034
42	0.67	1.95 (1.39–2.75)	<0.001	0.05	1.05 (0.64–1.73)	0.840	0.86	2.36 (1.53–3.65)	<0.001	0.58	1.78 (1.07–2.96)	0.026

^1^ EWGSOP2 = European Working Group on Sarcopenia in Older Persons. ^2^ Poisson Regression, statistical significance *p* < 0.05. RR = Relative Risk. CI = Confidence Interval.

## Data Availability

The original contributions presented in the study are included in the article, further inquiries can be directed to the corresponding author.

## Supplementary Materials

The following supporting information can be downloaded at: https://www.mdpi.com/article/10.3390/nu16111717/s1, Table S1: Outcomes observed follow-up time sarcopenia screening using EWGSOP tool; Outcomes observed follow-up time sarcopenia screening using SARCF11tool; Outcomes observed follow-up time sarcopenia screening using SARCF4 tool.
