# Supplementary material for: Prognostic Value of New Sarcopenia Screening Tool in the Elderly—SARC-GLOBAL
Source: Nutrients. 2024 May 31;16(11):1717. doi: 10.3390/nu16111717 (PMC11175117; doi:10.3390/nu16111717)
Supplement: Supplementary file 1 [file nutrients-16-01717-s001.zip › nutrients-2978862-supplementary.pdf]

Supplementary Materials

Prognostic Value of New Sarcopenia Screening Tool in the Elderly– SARC-GLOBAL

Ana Carolina Costa Vicedomini [1], Dan Linetzky Waitzberg [1], Natalia Correia Lopes [1], Natalia Magalhães [1], Ana Paula Aguiar Prudêncio [1], Wilson Jacob [2], Alexandre Busse [2], Douglas Ferdinando [2], Tatiana Pereira Alves [2], Rosa Maria Rodrigues Pereira [3] and Giliane Belarmino [1]

Table S1. Outcomes observed follow-up time sarcopenia screening using EWGSOP tool.

Follow-up time (months)	EWGSOP Sarcopenic				EWGSOP Non Sarcopenic			
	12	24	36	42	12	24	36	42
Clinic Outcomes								
Falls	14 (16,47)	5 (6,94)	1 (1,49)	4 (6,45)	53 (17,1)	16 (5,99)	6 (2,5)	5 (2,15)
Fractures	1 (1,18)	6 (8,33)	4 (5,97)	3 (4,84)	2 (0,65)	12 (4,49)	6 (2,5)	13 (5,58)
Infections	16 (18,82)	6 (8,33)	4 (5,97)	3 (4,84)	53 (17,1)	12 (4,49)	6 (2,5)	13 (5,58)
Hospitalizations	12 (14,12)	5 (6,94)	3 (4,48)	1 (1,61)	34 (10,97)	15 (5,62)	4 (1,67)	7 (3)
Death	2 (2,35)	1 (1,39)	1 (1,49)	0 (0)	5 (1,61)	3 (1,12)	0 (0)	1 (0,43)
Total	85	72	67	62	310	267	240	233

Outcomes observed follow-up time sarcopenia screening using SARCF11tool.

Follow-up time (months)	SARCF11 Sarcopenic				SARCF11 Non Sarcopenic			
	12	24	36	42	12	24	36	42
Clinic Outcomes								
Falls	9 (16,98)	2 (4,65)	0 (0)	2 (5,56)	58 (16,96)	19 (6,42)	7 (2,61)	7 (2,7)
Fractures	2 (3,77)	3 (6,98)	3 (7,69)	2 (5,56)	1 (0,29)	15 (5,07)	7 (2,61)	14 (5,41)
Infections	10 (18,87)	3 (6,98)	3 (7,69)	2 (5,56)	59 (17,25)	15 (5,07)	7 (2,61)	14 (5,41)
Hospitalizations	9 (16,98)	5 (11,63)	2 (5,13)	1 (2,78)	37 (10,82)	15 (5,07)	5 (1,87)	7 (2,7)
Death	2 (3,77)	2 (4,65)	0 (0)	0 (0)	5 (1,46)	2 (0,68)	1 (0,37)	1 (0,39)
Total	53	43	39	36	342	296	268	259

Outcomes observed follow-up time sarcopenia screening using SARCF4 tool.

Follow-up time (months)	SARCF4 Sarcopenic				SARCF4 Non Sarcopenic			
	12	24	36	42	12	24	36	42
Clinic Outcomes								
Falls	27 (38,03)	4 (6,25)	2 (3,28)	3 (5,17)	40 (12,35)	17 (6,18)	5 (2,03)	6 (2,53)
Fractures	1 (1,41)	5 (7,81)	3 (4,92)	4 (6,9)	2 (0,62)	13 (4,73)	7 (2,85)	12 (5,06)
Infections	12 (16,9)	5 (7,81)	3 (4,92)	4 (6,9)	57 (17,59)	13 (4,73)	7 (2,85)	12 (5,06)
Hospitalizations	14 (19,72)	8 (12,5)	3 (4,92)	3 (5,17)	32 (9,88)	12 (4,36)	4 (1,63)	5 (2,11)

Death	0 (0)	1 (1,56)	1 (1,64)	1 (1,72)	7 (2,16)	3 (1,09)	0 (0)	0 (0)
Total	71	64	61	58	324	275	246	237
